# The status and trends of type 2 diabetic osteoporosis research: a global bibliometric and visualization analysis over the past 20 years

**DOI:** 10.3389/fcdhc.2025.1596938

**Published:** 2025-06-10

**Authors:** Haiyan Hou, Liying Zhu

**Affiliations:** Department of Infectious Diseases, The Second Affiliated Hospital of Harbin Medical University, Harbin Medical University, Harbin, Heilongjiang, China

**Keywords:** type 2 diabetes mellitus, osteoporosis, bibliometrics, VOSviewer, hotspots, global health trends, diabetes complications

## Abstract

**Background:**

Type 2 diabetic osteoporosis (T2DOP) has received considerable attention due to its accelerated bone deterioration and significantly increased fracture risk. Unlike classical osteoporosis, patients with T2DOP often exhibit a paradoxical pattern: they have normal or even elevated bone mineral density (BMD) in early stages despite deterioration in bone microarchitecture. This paradox highlights the clinical importance of identifying T2DOP as a distinct and critical subtype of secondary osteoporosis.

**Methods:**

We conducted a bibliometric analysis of literature on T2DOP published over the past 20 Years(from 2001 to 2020), using data retrieved from the Web of Science Core Collection database. Bibliometric networks were visualized and analyzed using VOSviewer. Publication trends, geographic contributions, research hotspots, and keyword clusters were systematically examined.

**Results:**

Over the past 20 Years, global research output on T2DOP steadily increased, with major contributions from North America, East Asia, and Western Europe. Identified research hotspots included risk prediction, biomarkers (e.g., advanced glycation end-products), complication management, population-specific characteristics (e.g., postmenopausal women), and therapeutic strategies (e.g., metformin). Notably, lifestyle intervention has recently emerged as an important new research direction.

**Conclusions:**

This study provides the first comprehensive bibliometric analysis and visualization of global research trends and hotspots in T2DOP, highlighting critical insights for clinical practice, including the identification of at-risk populations, biomarker-guided risk assessment, and therapeutic optimization, which complements existing clinical meta-analyses. Future research efforts should emphasize multidisciplinary collaboration and validation of the long-term efficacy of lifestyle interventions. For clinical practice, integrating bone density evaluation with biomarker screening (e.g., osteocalcin) in diabetic patients could enhance early fracture prevention. Public health initiatives should prioritize lifestyle interventions in high-risk populations (e.g., postmenopausal women) to mitigate the growing burden of diabetic osteoporosis.

## 1 Introduction

Type 2 diabetes mellitus (T2DM) represents a major global health challenge, particularly in Asia. Notably, China has the largest diabetic population worldwide, with 141 million adults aged 20–79 living with diabetes, as reported in the IDF Diabetes Atlas (10th edition, 2021) (1). Approximately 50%–66% of individuals with diabetes exhibit reduced bone mineral density (BMD), and nearly one-third meet the diagnostic criteria for osteoporosis. Consequently, individuals with diabetes have a 2- to 3-fold higher risk of fractures than those without diabetes (2). Chronic hyperglycemia contributes to the development of T2DOP through mechanisms such as the accumulation of advanced glycation end-products (AGEs), increased oxidative stress, and inhibition of the Wnt/β-catenin signaling pathway. These pathways collectively lead to impaired bone metabolism and are recognized as key factors in the pathogenesis of diabetic osteoporosis (3). Type 2 diabetic osteoporosis (T2DOP) is often characterized by paradoxically normal or increased bone mineral density (BMD) at diagnosis; however, this elevated BMD does not reflect improved bone quality and instead corresponds to a higher fracture risk. Consequently, T2DOP has become a crucial area within secondary osteoporosis research. Although osteoporosis in type 2 diabetes has been extensively investigated, comprehensive studies evaluating the development and trends within this research area are lacking. In addition, traditional systematic reviews are limited by qualitative assessments and cannot fully capture complex research landscapes or emerging trends. Bibliometric analysis is particularly suited to overcome these limitations by quantitatively identifying research hotspots, clarifying knowledge gaps, and visualizing collaboration networks. Thus, we conducted a bibliometric analysis of literature on osteoporosis associated with T2DM (2001–2020) to systematically map the research landscape, address existing knowledge gaps, and identify evolving trends clearly (4, 5).

## 2 Materials and methods

### 2.1 Retrieval strategy and data source

**Figure 1:**
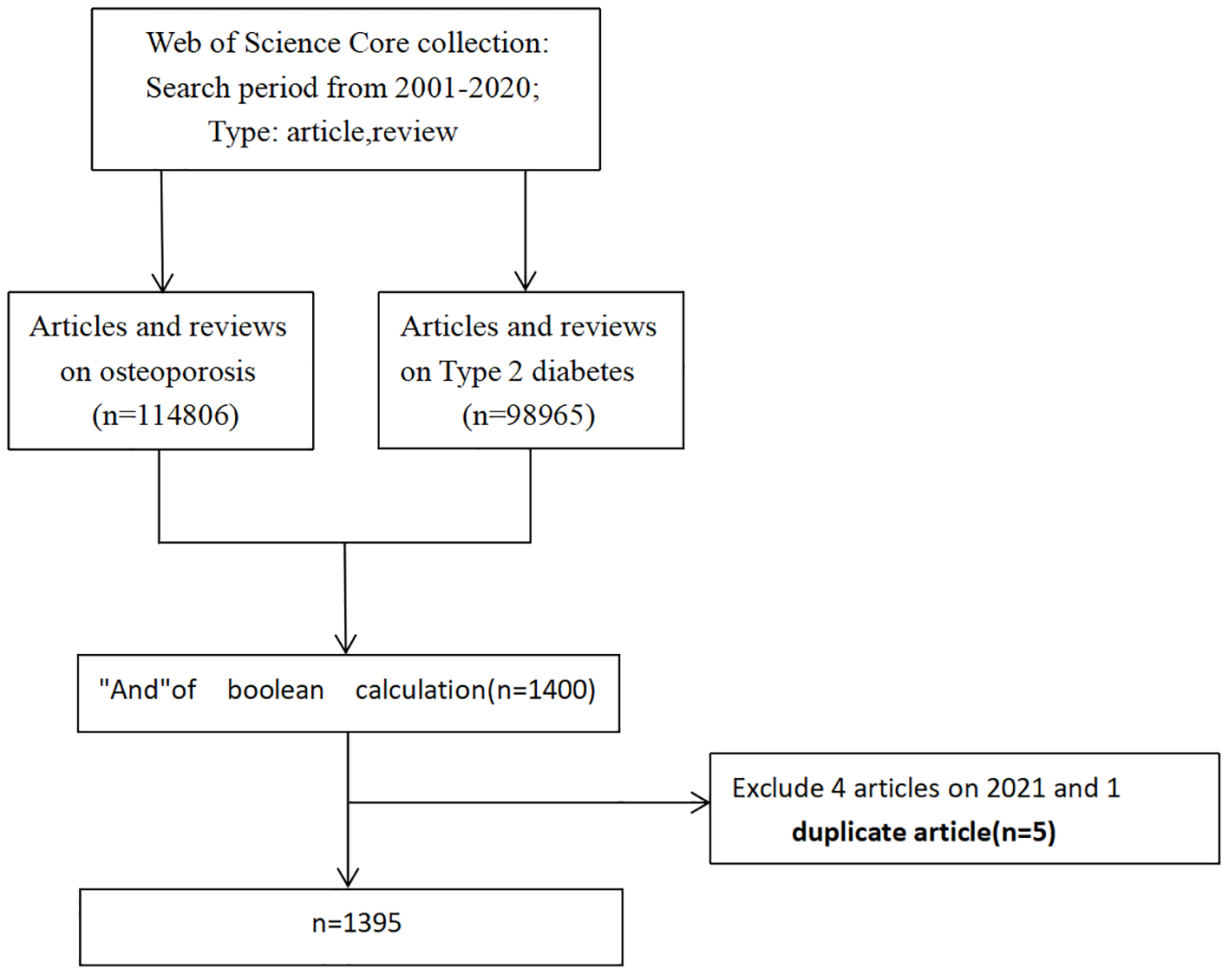
Flow chart of inclusion and exclusion criteria.

We conducted a systematic bibliometric analysis of literature related to osteoporosis in type 2 diabetes mellitus (T2DM) published from 2001 to 2020. Publications were retrieved exclusively from the Web of Science Core Collection (WOSCC), including SCI-Expanded, SSCI, A&HCI, CPCI-S, ESCI, CCR-Expanded, and IC. WOSCC was selected due to its structured indexing, compatibility with bibliometric software (e.g., VOSviewer), and widespread use in high-quality bibliometric studies. Although other databases such as PubMed or Scopus were not included, the use of WOSCC ensures data consistency and enables effective mapping of citation and co-authorship networks. The search query used was: TS = ((“type 2 diabetes” OR T2DM OR “non-insulin dependent diabetes”) AND (“osteoporosis”)). After the initial retrieval, both duplicate entries and irrelevant publications were excluded, resulting in a final dataset of 1,395 articles (see **Figure 1** for the selection flow chart), which were analyzed using VOSviewer.

### 2.2 Data collection

Bibliographic records from the Web of Science Core Collection were exported and managed in Microsoft Excel 2021 for initial data organization and screening.

### 2.3 Statistical analysis

First, Microsoft Excel 2021 was utilized to categorize and process the collected literature, followed by data mining to analyze publication trends, journals, collaboration, co-citation and research hotspots.

VOSviewer, a widely used software for bibliometric analysis and data visualization, was employed to identify highly cited publications, leading journals, key contributing countries, institutions, and prominent authors along with their research collaborations. Additionally, co-citation network analysis was performed on references and authors to elucidate the foundational research in this field.

Cluster analysis was conducted using VOSviewer’s built-in Louvain algorithm, with a resolution parameter set at 1.0. To ensure thematic coherence, clusters containing fewer than five items were excluded, following best practices in bibliometric network analysis (6). To validate the interpretability and coherence of these clusters, we examined the constituent keywords within each cluster and found that the items in each group shared clear thematic commonalities. For example, one cluster included many terms related to a particular sub-topic, whereas another cluster was characterized by terms pertaining to a different theme, indicating that each cluster represents a distinct area of research. This semantic coherence suggests that the clustering results are meaningful and consistent with domain knowledge. In the VOSviewer diagram, each color represents a distinct thematic cluster. Node size reflects occurrence frequency; therefore, larger nodes indicate higher frequency. Lines connecting nodes illustrate co-occurrence relationships, with thicker lines signifying stronger associations. Consequently, larger nodes and thicker connecting lines denote higher frequency and stronger co-occurrence, respectively.

## 3 Results

### 3.1 Publication outputs analysis

**Figure 2:**
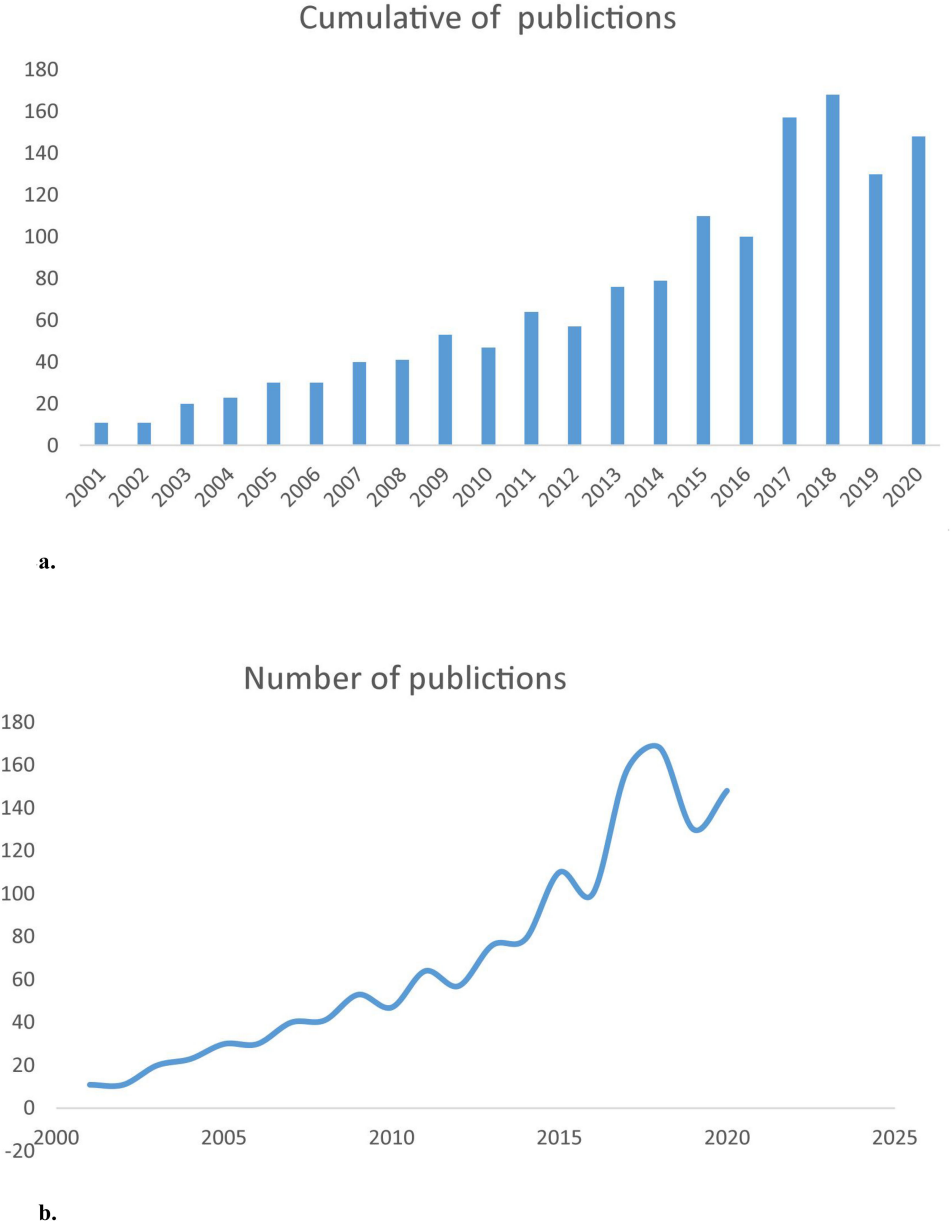
**(a)** The annual number of publication on T2DOP research from 2001 to 2020. **(b)** The curve fitting of publication growth trend of T2DOP research.

Microsoft Excel was used to analyze and visualize publication trends from 2001 to 2020. The number of publications showed a consistent upward trend over the past two decades, increasing from fewer than 30 articles in 2001 to nearly 150 in 2020 (**Figure 2a**), corresponding to an average annual growth rate of approximately 7%. Although slight fluctuations occurred—most notably minor declines in 2018 and 2019—the overall trajectory reflects steadily growing research interest in osteoporosis associated with type 2 diabetes (**Figure 2b**).

### 3.2 Journals, collaboration and co-citation analysis

**Table 1** presents the top 20 journals ranked by the number of publications in the field of type 2 diabetic osteoporosis. These journals collectively published 386 articles, representing approximately 27.67% of the total publications analyzed. *Osteoporosis International* was the most productive journal (51 articles, 3.65%), while the *Journal of Bone and Mineral Research* had the highest impact factor (5.853). Notably, the *Journal of Clinical Endocrinology & Metabolism* ranked second in IF (5.399) while also demonstrating strong academic influence by citation metrics.

**Table 1:** The top 20 most active journals that published articles on T2DOP research from 2001 to 2020.

Journal	Published items	Citations	Average citations per item	IF
Osteoporosis International	51	2673	52.41	3.864
Journal of Clinical Endocrinology & Metabolism	44	4678	106.31	5.399
Bone	38	1082	28.47	4.147
Journal of Bone and Mineral Research	29	2549	87.89	5.854
Journal of Periodontology	26	945	36.34	3.742
Calcified Tissue International	24	772	32.16	2.297
Journal of Bone and Mineral Metabolism	24	276	11.5	3.423
Plos One	22	221	10.04	2.74
Journal of Clinical Densitometry	14	173	12.35	2.31
Diabetes Care	13	1415	108.84	2.052
Diabetes Research and Clinical Practice	13	217	16.69	4.234
Endocrine	13	208	16	3.235
Endocrinology	12	709	59.08	3.934
Clinical Oral Implants Research	11	199	18.09	3.723
Diabetic Medicine	11	211	19.18	3.083
Journal of Diabetes Investigation	11	59	5.36	3.761
Frontiers in Endocrinology	10	193	19.3	3.644
Journal of Diabetes and its Complications	10	183	18.3	2.781
Maturitas	10	177	17.7	3.63
Acta Diabetologica	9	186	20.66	3.418

IF, Impact fact, Using the 2019 IF as the standard.

**Figure 3:**
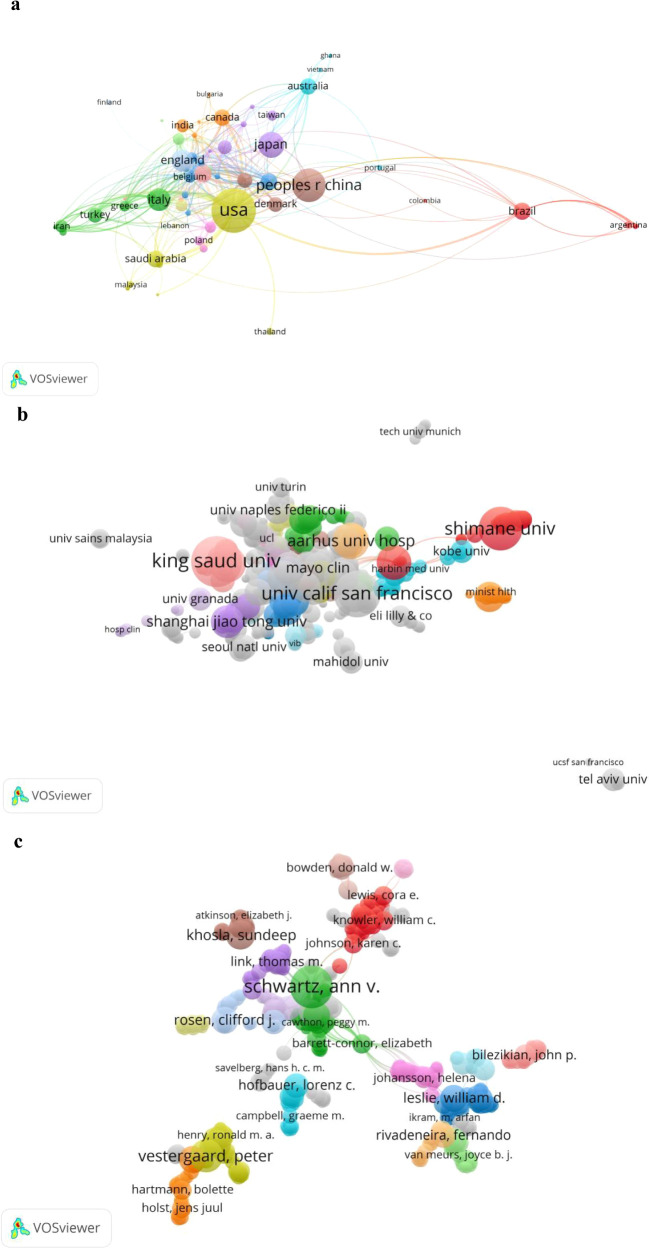
**(a)** Network visualization map of countries collaboration in T2DOP research during the period 2001 to 2020. **(b)** Network map of collaboration between institutions. **(c)** Network map of collaboration between authors. (The size of the nodes is related to the number of articles they publish, the link between two nodes indicates that they have collaborations, and the link strength indicates the strength of collaboration between countries/institutions/authors, the strength number is displayed only on VOSviewer).

In terms of collaboration, a total of 81 countries contributed to the 1,395 publications analyzed in this study. Among these, the United States was the leading contributor with 370 publications (20%), followed by China (210 publications, 12%) and Japan (125 publications, 7%). In terms of total citations, the United States ranked first with 25,935 citations (**Table 2**), followed by Canada with 7,022 citations, indicating that Canadian publications, though fewer in number, were cited often. Notably, Canada exhibited the highest average citation count per article despite fewer publications, indicating its substantial research impact. Additionally, China’s lower total citation count compared to the U.S. may be attributed to language barriers, preference for local Chinese-language journals, limited international collaboration, and relatively recent publication dates (shorter citation accumulation period), rather than merely indicating a quality gap. Scientific collaboration networks were further visualized to assess inter-country cooperation in the T2DOP field (**Figure 3a**). The United States occupied the central position in the global collaboration network, characterized by the largest node size and the highest number of connections, highlighting its influential role and extensive research interactions internationally. Specifically, the strongest collaboration was observed between China and the United States, with a connection strength of 25, demonstrating frequent academic exchanges and close research cooperation between these two countries.

**Table 2:** The top 10 country producing articles on T2DOP research by record count.

Rank	Country	Publications	Received citations	Average citations per item
1	USA	370	25935	70.09
2	China	210	2154	10.25
3	Japan	125	3334	26.67
4	Italy	97	2899	29.20
5	England	88	3749	42.60
6	Germany	56	3972	70.92
7	Brazil	55	1565	28.45
8	Saudi Arabia	54	733	13.57
9	Spain	52	1609	30.94
10	Canada	50	7022	140.44

In terms of institutional contributions, King Saud University in Saudi Arabia ranked first among the top 20 institutions (**Table 3**), with the highest number of publications (39 articles). King Saud University had a total of 68 collaboration links (**Figure 3b**), although most of these were weaker connections (indicating limited collaboration intensity with any single partner). The University of California (USA), despite having fewer publications (33), exhibited broader collaboration, demonstrated by a higher number of connections (83) and greater total connection strength (145).

**Table 3:** The top 10 organization producing articles on T2DOP research by record count.

Rank	Institutional	Document	Citation	Average citations per item
1	King Saud Univ	39	550	14.10
2	Univ Calif San Francisco	33	3447	104.45
3	Shimane Univ	29	1440	49.66
4	Aarhus Univ Hosp	20	1958	97.90
5	Univ Rochester	20	381	19.05
6	Univ Minnesota	18	4209	233.83
7	Univ Pittsburgh	18	2485	138.06
8	Univ Sao Paulo	17	753	44.29
9	Shanghai Jiao Tong Univ	16	254	15.88
10	Univ Copenhagen	16	1942	121.38

**Figure 4:**
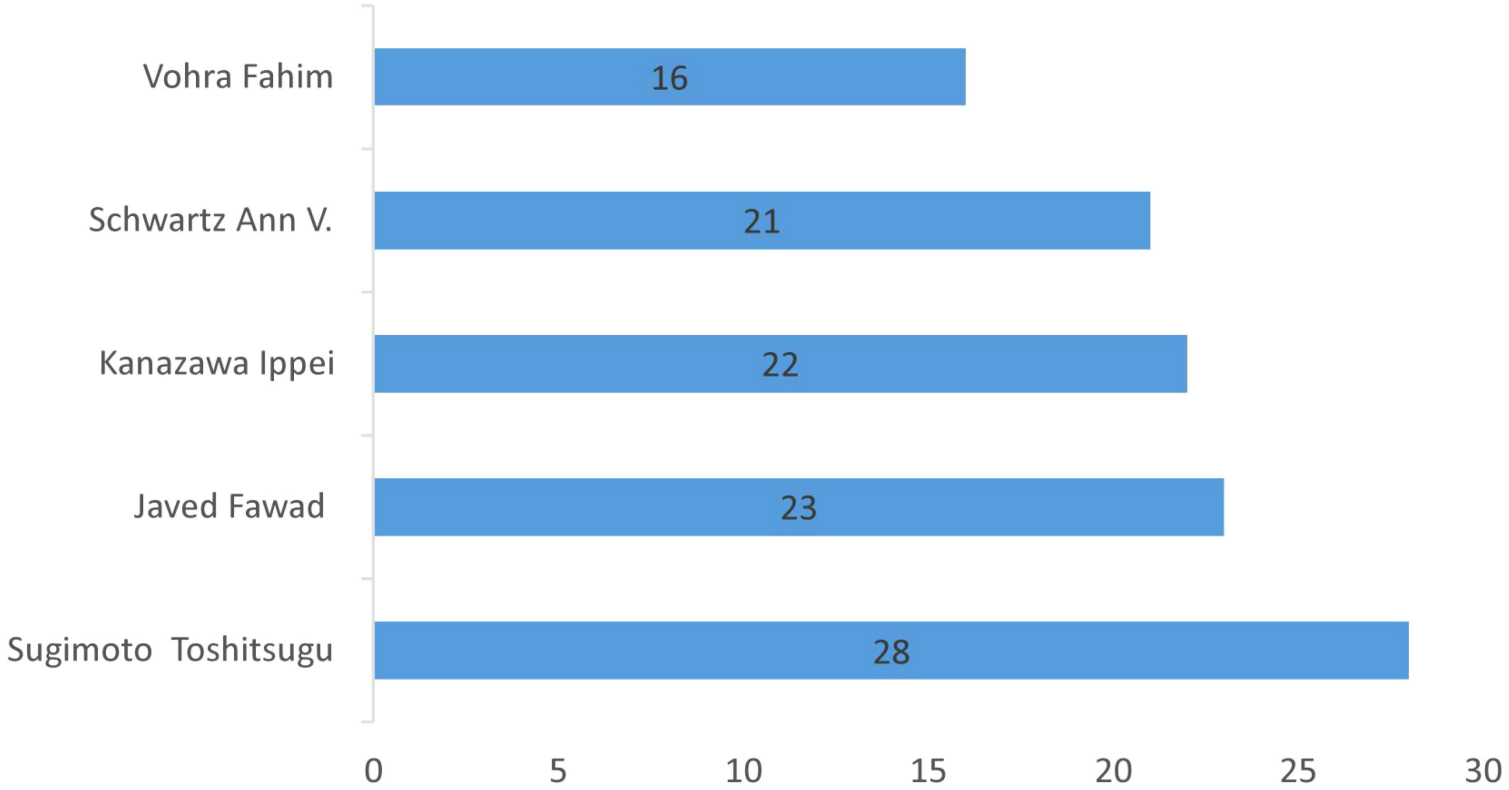
The top 5 author producing articles on T2DOP research by record count.

With respect to authorship, a total of 7,305 authors contributed to the 1,395 publications on type 2 diabetic osteoporosis. **Figure 3c** illustrates author collaboration networks. **Figure 4** displays the top five contributing authors in the T2DOP study articles, highlighting the most active researchers. Sugimoto Toshitsugu (Shimane University, Japan) published the highest number of articles (28) with the greatest total connection strength (2360). Javed Fawad from the University of Rochester (USA) ranked second with 22 publications and a total connection strength of 954, while Kanazawa Ippei from Shimane University (Japan) ranked third with 18 publications and a total connection strength of 1206. Schwartz Ann V. from the USA ranked fifth, showing the highest total connection strength (1979) among leading authors, indicative of extensive research collaboration. Additionally, although Willett Walter C. authored only two publications, his high connection strength (1979) and significant citation count (1,079 citations) underscore his extensive collaboration and strong academic influence in the field.

**Figure 5:**
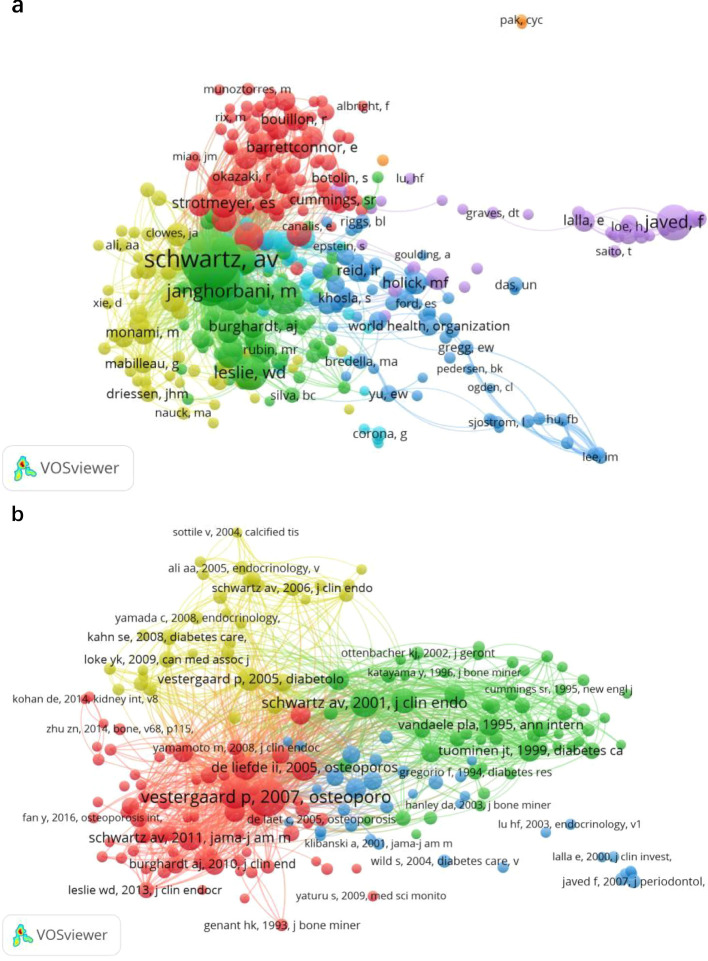
**(a)** Mapping of co-cited authors related to T2DOP. **(b)** Mapping of co-cited references related to T2DOP. (A line between two nodes means that both are cited in the same paper, and a shorter line indicates a stronger connection between the two).

Co-citation analysis measures the relationship between different publications based on how frequently they are cited together. As shown in **Figure 5a**, the most co-cited paper was a meta-analysis published in Osteoporosis International (2007), titled “Discrepancies in bone mineral density and fracture risk in patients with type 1 and type 2 diabetes,” cited 281 times. The systematic review “Systematic review of type 1 and type 2 diabetes mellitus and risk of fracture,” published by Janghorbani M. in 2007, ranked second, with 197 citations. Among the most co-cited authors (**Figure 5b**), Schwartz AV (653 citations), Vestergaard P (483 citations), and Janghorbani M (257 citations) were identified as leading researchers, indicating their significant influence and foundational roles in this research area.

### 3.3 Keywords analysis

**Figure 6:**
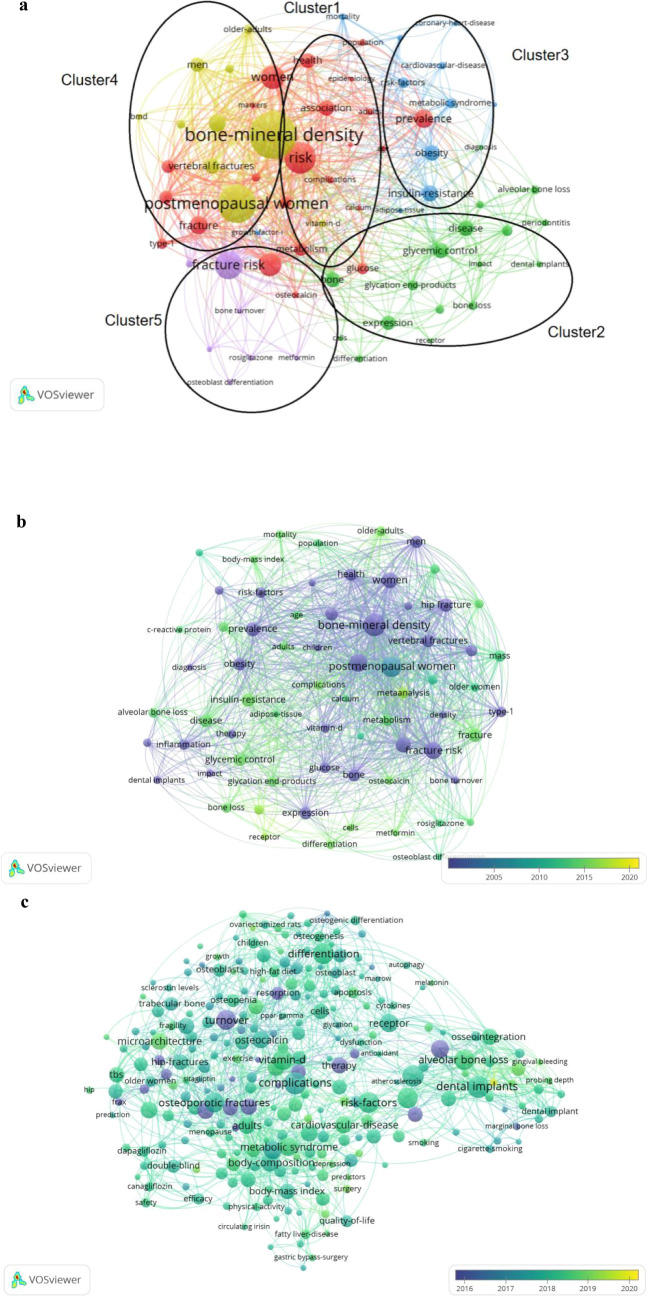
**(a)** Overlay visualization map of keywords occurrences. [The size of the nodes represented the frequency, and the nodes with the same color belonged to the same cluster. The keywords were divided into four clusters: Risk study (cluster 1), biomarker study (cluster 2), complication study (cluster 3) and population study (cluster 4), and treatment study (cluster 5)]. **(b)** The time distribution of keywords according to the mean frequency of appearance from 2001-2020. (The blue keywords appeared earlier and the yellow keywords later). **(c)** The time distribution of keywords according to the mean frequency of appearance from 2016 to 2020. (The blue keywords appeared earlier and the yellow keywords later).

Keywords provide a highly condensed representation of literature content, and keyword co-occurrence analysis effectively highlights major research hotspots. A total of 1,395 articles related to type 2 diabetic osteoporosis were analyzed using VOSviewer with a keyword occurrence threshold set at 30. After merging synonymous keywords and excluding the highly frequent but non-informative terms (“type 2 diabetes,” “T2DM,” and “osteoporosis”), five distinct thematic clusters emerged, as shown in **Figure 6a**. These clusters included “Risk Study,” “Biomarker Study,” “Complication Study,” “Population Study,” and “Therapeutic Study”.

In the *Risk Study* cluster, prominent keywords included “risk” (190 occurrences), “fracture” (109 occurrences), “hip fracture” (106 occurrences), and “vertebral fracture” (94 occurrences).The *Biomarker Study* cluster featured keywords such as “advanced glycation end-products” (61 occurrences) and “osteocalcin” (42 occurrences).In the *Complication Study* cluster, frequently occurring keywords were “obesity” (93 occurrences), “dental implants” (52 occurrences), “cardiovascular disease mortality” (41 occurrences), and “coronary heart disease” (32 occurrences).The *Population Study* cluster was characterized by keywords including “bone mineral density” (707 occurrences), “postmenopausal women” (232 occurrences), “men” (87 occurrences), and “older adults” (58 occurrences).Within the *Therapeutic Study* cluster, “thiazolidinediones” (34 occurrences), “rosiglitazone” (34 occurrences), and “metformin” (30 occurrences) emerged as central keywords.

**Figures 6b, c** illustrates the temporal evolution of keyword trends, with earlier keywords appearing in blue and more recent ones in yellow. Initially, research concentrated on risk factors and population characteristics, notably “risk,” “fracture,” “postmenopausal women,” and “older adults.” Subsequently, keywords such as “thiazolidinediones” and “metformin” within therapeutic studies and “advanced glycation end-products” within biomarker studies gained greater attention. More recently, emerging keywords in the past five years included “quality-of-life” (13 occurrences) and “lifestyle intervention” (5 occurrences), indicating their growing relevance in this research area.

## 4 Discussion

Bibliometric analysis has been widely recognized as an effective approach for systematically mapping current research landscapes and identifying emerging research hotspots (7–9). VOSviewer is a widely used bibliometric visualization tool that effectively provides comprehensive insights into current research trends and evolving hotspots within type 2 diabetes-related osteoporosis by visualizing publication networks and thematic clusters.

### 4.1 Publication outputs analysis

Over the past 20 years, research output on T2DOP has steadily grown, rapidly increasing between 2014 and 2018, peaking in 2018 (168 publications). Despite significant growth, T2DOP remains a challenging clinical issue, necessitating continued research.

### 4.2 Journals, collaboration and co-citation analysis

Journal analysis highlights Osteoporosis International and the Journal of Clinical Endocrinology & Metabolism as significant contributors to T2DOP research. The United States, China, and Japan are major contributing countries, with the U.S. exhibiting extensive international collaboration. Key institutions include King Saud University and the University of California. Co-citation analysis identifies Schwartz AV, Vestergaard P, and Janghorbani M as influential researchers who substantially advanced understanding of bone fragility and therapeutic mechanisms. Overall, international and institutional collaborations are crucial for the advancement of T2DOP research.

### 4.3 Hotspot

VOSviewer was used to analyze key keywords related to osteoporosis research in type 2 diabetes, providing insight into emerging research trends and frontiers. Beyond high-frequency keywords such as “type 2 diabetes,” “diabetes,” and “osteoporosis,” each keyword cluster contains valuable terms that warrant attention for understanding research priorities in this field.

#### 4.3.1 Cluster 1 (Risk)

Analysis of Cluster 1 (Risk) reveals that scholars place significant emphasis on the fracture risk associated with type 2 diabetes. This cluster highlights the increasing concern over bone fragility and fracture susceptibility in diabetic populations, reinforcing the necessity of risk assessment and targeted preventive strategies. As fracture risk remains a major clinical challenge in T2DOP, future research is expected to focus on improving risk prediction, identifying high-risk individuals, and developing targeted interventions. Indeed, studies show that individuals with T2DM have a 2- to 3-fold higher risk of osteoporotic fractures than non-diabetics, despite often paradoxically normal or even elevated BMD. This suggests diabetes compromises bone quality and microarchitecture rather than bone mass, underscoring the importance of early screening and preventive strategies (3, 10).

This increased fracture risk in individuals with type 2 diabetes is primarily due to abnormal bone remodeling, characterized by elevated blood glucose levels, accumulation of advanced glycation end-products (AGEs), defects in terminal product processing, altered insulin levels or function, reduced insulin-like growth factor 1 (IGF-1) levels, oxidative stress, and increased proinflammatory cytokines. These factors contribute to osteoblast and osteoclast dysfunction, ultimately leading to impaired bone quality and an increased susceptibility to fractures.

Compared to individuals without diabetes, patients with type 2 diabetes have a 20% higher risk of fractures at any site, and their risk of hip fracture later in life is 2.7 times higher than that of the general population (11), The rate of vertebral fractures in patients with type 2 diabetes was 2.03 times higher than that in non-diabetic individuals (12). Notably, vertebral (spine) fractures and hip fractures are particularly devastating in T2DOP. The spine bears most of the body’s weight and is prone to fracture under diabetic bone fragility, and hip fractures in older diabetic patients carry high morbidity and up to ~20% one-year mortality—earning the moniker “the final fracture.

Understanding the fracture risk associated with different skeletal sites in T2DM is crucial for the prevention and management of T2DOP. Effective strategies to mitigate fracture risk in T2DM patients can help reduce complications and improve long-term clinical outcomes.

#### 4.3.2 Clusters 2 and 4 (biomarker, population)

Keywords related to pathogenesis, such as advanced glycation end-products (AGEs), osteocalcin, postmenopausal women, and bone mineral density, were frequently identified in these clusters. Advanced glycation end-products (AGEs) are formed when proteins (amino acids) bind to sugars in the body through non-enzymatic glycation reactions. Individuals with diabetes experience accelerated AGE formation due to chronic hyperglycemia, which increases non-enzymatic glycation of proteins. In the skeletal system, AGEs negatively impact collagen fiber deposition in the bone matrix, reducing bone toughness and elasticity. This deterioration compromises bone mechanical properties and increases fracture risk in patients with diabetes (13). The role of advanced glycation end-products (AGEs) in bone fragility highlights their significance as a key biomarker in the study of T2DOP and underscores the necessity of targeted therapeutic strategies to mitigate their detrimental effects on bone health. Another high-frequency keyword, osteocalcin, is the largest non-collagenous protein in bone and serves as the most sensitive and specific biomarker of osteoblastic activity. As a critical regulator of bone formation and metabolism, osteocalcin plays an essential role in maintaining bone strength and structural integrity. Monitoring osteocalcin levels can provide valuable insights into bone turnover and osteoblast function, making it an important diagnostic and prognostic marker in T2DOP research (14, 15). The serum (plasma) N-MID osteocalcin level is closely associated with bone turnover rate, varying with both age and bone metabolism dynamics. Specifically, higher bone turnover rates correspond to elevated osteocalcin levels, while lower turnover rates result in reduced osteocalcin levels. The measurement of serum N-MID provides valuable insight into osteoblast activity, making it a reference index for diagnosing osteoporosis syndrome and an important biomarker for assessing bone metabolism in the elderly.

Studies have shown that in primary osteoporosis, postmenopausal women (42–84 years old) exhibit increased N-MID levels, primarily due to estrogen deficiency. In men (54–88 years old), N-MID elevation is mainly attributed to age-related changes in bone metabolism. Currently, serum (plasma) osteocalcin (N-MID) is widely recognized as a biomarker of bone formation and is commonly used to evaluate the efficacy of anti-resorptive therapies for conditions such as osteoporosis and hypercalcemia.

Bone markers, including N-MID osteocalcin, can reflect systemic changes in bone metabolism and provide early insights into treatment efficacy and patient compliance within 3 to 6 months. When combined with bone mineral density (BMD) assessments, these markers serve as important diagnostic indicators for osteoporosis, aiding in both early detection and therapeutic monitoring (16). The detection of biomarkers in individuals with type 2 diabetes can play a crucial role in the early prevention and management of osteoporosis. By monitoring key bone metabolism markers, such as osteocalcin (N-MID) and advanced glycation end-products (AGEs), clinicians can assess bone turnover status, identify high-risk individuals, and implement timely interventions to mitigate fracture risk. Early biomarker-based screening strategies can improve osteoporosis prevention efforts and enhance treatment outcomes in patients with type 2 diabetes.

#### 4.3.3 Cluster 3 (complications)

As the duration of diabetes increases, the incidence of complications rises significantly. According to a 2010 report by the American Diabetes Association (ADA), the prevalence of complications among diabetic patients is strongly correlated with the disease duration. According to an ADA report, complication prevalence rises with diabetes duration: >46% of patients develop at least one complication within 3 years of T2DM onset; >61% within 5 years; and as high as 98% after 10 years. These findings emphasize the progressive nature of diabetes-related complications, reinforcing the need for early intervention, continuous monitoring, and comprehensive management strategies to reduce long-term health risks in diabetic patients (17). In addition to osteoporotic fractures, type 2 diabetes is associated with over 100 complications, including cardiovascular disease and kidney disease. However, oral diseases, particularly periodontitis, are often overlooked as a significant diabetic complication.

Studies have reported that patients with diabetes are two to three times more likely to develop severe chronic periodontitis compared to non-diabetic individuals. Moreover, alveolar bone resorption and attachment loss are significantly more severe in diabetic patients than in those with ordinary periodontitis. The incidence of diabetes-related periodontitis increases with age and disease duration, highlighting the need for early oral health screening and preventive interventions in diabetic patients (18). These oral complications in diabetic patients, including gingival atrophy, swelling, pain, periodontal infections, bad breath, and tooth loosening or loss, are likely associated with long-term poor blood sugar control. Chronic hyperglycemia can lead to impaired immune function, increased susceptibility to secondary infections, and excessive calcium loss, all of which contribute to the progression of periodontal disease in diabetic individuals.

The primary danger of diabetes lies in its wide range of complications, making prevention more critical than treatment. Early intervention is essential, as timely management significantly reduces the risk and severity of complications compared to delayed treatment.

#### 4.3.4 Cluster 5 (treatment)

The high-frequency keywords in the treatment cluster include “thiazolidinediones,” “metformin,” and “older women”, highlighting key therapeutic concerns in the management of type 2 diabetic osteoporosis (T2DOP).

Thiazolidinediones (TZDs), particularly rosiglitazone, are associated with increased fracture risk in diabetic patients, especially among older women. The experiment demonstrates that TZDs may alter bone remodeling by shifting mesenchymal stem cell differentiation toward adipocytes rather than osteoblasts (19). Additionally, studies have demonstrated that older women using thiazolidinediones (TZDs) have significantly elevated fracture risks, with risks further stratified by increasing age and longer treatment duration. For instance, Schwartz et al. reported that postmenopausal women treated with TZDs for extended periods (≥2 years) exhibited notably higher fracture risk compared to short-term users (20). This indicates the necessity of risk stratification in clinical practice, recommending regular bone density monitoring and preventive interventions for high-risk elderly women receiving TZDs.

Metformin is another hypoglycemic drug that has received a lot of attention. Clinical studies have shown that metformin can protect bone health and reduce fracture risk in diabetic patients (21–23). The mechanism underlying metformin’s protective effects on bone health is related to its ability to activate the AMPK (adenosine monophosphate-activated protein kinase) signaling pathway. This activation induces osteoblast differentiation and mineralization, thereby promoting bone formation and potentially reducing the risk of fractures in diabetic patients (24, 25). Metformin is a first-line hypoglycemic agent for the treatment of type 2 diabetes and has also been recognized for its potential benefits in osteoporosis management (26). At present, T2DOP has become a significant health concern. When formulating individualized treatment plans for patients with diabetes-associated osteoporosis, it is essential to fully consider the effects of hypoglycemic drugs on bone health. Additionally, anti-osteoporosis medications should be co-administered when appropriate to ensure comprehensive management and reduce fracture risk.

Keywords can also be used to track research trends in type 2 diabetic osteoporosis (T2DOP), as shown in **Figures 6b, c**. Early research in this field centered on fracture risk assessment and population-based studies (*diagnosis* and epidemiology). As the field matured, the focus shifted toward *treatment* strategies and biomarkers, as well as the prevention and management of complications. In the past five years, with diabetes prevalence rising and patients living longer, there is growing emphasis on non-pharmacological approaches; indeed, “lifestyle intervention” has emerged as a notable keyword (reflecting interest in exercise, diet, and other modifications to manage T2DOP). This trend is logical given that T2DOP’s high disability and economic burden make rehabilitation challenging – preventing complications through early lifestyle changes is therefore crucial to improving patient outcomes. In summary, the evolution of keywords illustrates that T2DOP research priorities have shifted over time: earlier work focused on fracture risk assessment and population epidemiology, then attention moved toward treatment strategies, biomarkers, and the management of complications. In the past five years, as the number of diabetes patients has grown and they live longer, non-pharmacological approaches like “lifestyle intervention” have emerged as new hotspots. This shift aligns with clinical needs, because T2DOP is associated with high disability rates and economic burdens; thus, intervening early with lifestyle changes (such as exercise, diet) to prevent complications is crucial for improving long-term outcomes for these patients.

## 5 Limitations

Our study has some limitations. First, a recent systematic review and meta-analysis systematically quantified the clinical association between type 2 diabetes and osteoporosis-related fracture risks, using multiple databases including Web of Science, Embase, PubMed, and the Cochrane Library (27). In contrast, our study differs from this review in terms of objective, methodology, and application. While the systematic review aims to quantify clinical fracture risks, our study employs bibliometric analysis to investigate research trends, global collaboration patterns, and evolving hotspots in the field. Furthermore, the Web of Science is a multidisciplinary database of high-quality publications and is one of the most widely used sources for bibliometric analysis (28, 29). Furthermore, we limited our data to the Web of Science Core Collection to ensure broader coverage, higher-quality literature, and easier access to citation information, thereby enabling more accurate trend analysis (28, 30, 31). Finally, we focus on a 20-year window (2001–2020), providing a long-term perspective on scholarly output and thematic evolution, which is of particular value for research planning and policy-making, rather than direct clinical decision-making. We acknowledge the limitations related to database coverage and publication timeframe. Although we did not conduct sensitivity analyses across databases, we recommend that future bibliometric studies consider integrating multiple databases and extending the analysis period to enhance the robustness and applicability of our findings.

Second, all retrieved publications were in English, which may have introduced language bias and limited the inclusion of relevant studies published in other languages. This English-only focus may underrepresent research from non-English-speaking regions (e.g., Latin America, Russia, the Middle East), where significant findings are often published in local languages. This language bias could skew the interpretation of global research trends, as contributions from certain regions may be overlooked. We recommend that future bibliometric studies incorporate multilingual databases or regional indexing systems (e.g., SciELO for Latin American literature, CNKI for Chinese literature) to broaden geographic coverage and mitigate this bias.

Third, although the search criteria were carefully designed to define the research topic, we cannot guarantee that every included article is strictly focused on T2DOP. Despite these limitations, we believe that our study encompasses a sufficient number of publications from 2001 onward, providing a representative overview of the research landscape in this field. The small proportion of omitted data is unlikely to alter the overall findings, and our study remains a valuable tool for identifying general trends and developments in type 2 diabetic osteoporosis research.

## 6 Conclusion

This bibliometric analysis demonstrates that global research on T2DOP has steadily grown over the past two decades, with identified hotspots emphasizing risk assessment, biomarkers (e.g., advanced glycation end-products), complications, and therapeutic strategies. Importantly, lifestyle intervention has emerged as a critical frontier for future research. To translate these findings into clinical practice, we recommend integrating BMD screening with biomarker profiling (e.g., osteocalcin) could enhance fracture risk stratification. However, challenges like cost barriers must be addressed. Pilot models (e.g., Japan’s regional osteoporosis screening) may offer practical insights. Simultaneously, public health policymakers should prioritize community-based lifestyle intervention programs targeting postmenopausal women and older adults, who exhibit the highest vulnerability to T2DOP-related fractures. However, further validation of long-term intervention efficacy and international collaborative studies are needed to address geographic disparities in research output. These efforts will ultimately reduce the global burden of diabetic osteoporosis and improve patient outcomes through evidence-based prevention strategies.

## Data Availability

The original contributions presented in the study are included in the article/supplementary material. Further inquiries can be directed to the corresponding author/s.
